# ClonoCalc and ClonoPlot: immune repertoire analysis from raw files to publication figures with graphical user interface

**DOI:** 10.1186/s12859-017-1575-2

**Published:** 2017-03-11

**Authors:** Anke Fähnrich, Moritz Krebbel, Normann Decker, Martin Leucker, Felix D. Lange, Kathrin Kalies, Steffen Möller

**Affiliations:** 1 Institute of Anatomy, Ratzeburger Allee, Lübeck, 160, 23562 Germany; 2 Institute for Software Engineering and Programming Languages, Ratzeburger Allee, Lübeck, 160, 23562 Germany; 3 Institute for Biostatistics and Informatics in Medicine and Ageing Research, Ernst-Heydemann-Str. 8, Rostock, 18057 Germany

**Keywords:** Next-generation sequencing, Immune repertoire analysis, Graphical user interface

## Abstract

**Background:**

Next generation sequencing (NGS) technologies enable studies and analyses of the diversity of both T and B cell receptors (TCR and BCR) in human and animal systems to elucidate immune functions in health and disease. Over the last few years, several algorithms and tools have been developed to support respective analyses of raw sequencing data of the immune repertoire. These tools focus on distinct aspects of the data processing and require a strong bioinformatics background. To facilitate the analysis of T and B cell repertoires by less experienced users, software is needed that combines the most common tools for repertoire analysis.

**Results:**

We introduce a graphical user interface (GUI) providing a complete analysis pipeline for processing raw NGS data for human and animal TCR and BCR clonotype determination and advanced differential repertoire studies. It provides two applications. ClonoCalc prepares the raw data for downstream analyses. It combines a demultiplexer for barcode splitting and employs MiXCR for paired-end read merging and the extraction of human and animal TCR/BCR sequences. ClonoPlot wraps the R package tcR and further contributes self-developed plots for the descriptive comparative investigation of immune repertoires.

**Conclusion:**

This workflow reduces the amount of programming required to perform the respective analyses and supports both communication and training between scientists and technicians, and across scientific disciplines.

The Open Source development in Java and R is modular and invites advanced users to extend its functionality. Software and documentation are freely available at https://bitbucket.org/ClonoSuite/clonocalc-plot.

**Electronic supplementary material:**

The online version of this article (doi:10.1186/s12859-017-1575-2) contains supplementary material, which is available to authorized users.

## Background

Both B and T cells are essential for cellular immunity as key players in antigen recognition. The antigen, directly for B cells or presented as a cleaved fragment via the MHC for T cells, is bound by a cognate surface receptor. These B and T cell receptors have a well-defined sequence once the cell has matured. One distinguishes complementarity determining regions (CDR) 1 to 3 of which CDR3 is the most variable, but all contribute to antigen binding and need to be investigated together.

In order to provide protection from a wide range of pathogens, the diversity of these sequences is enormous. First measurements have estimated a mouse TCR diversity of 10^10^, which, however, is still short of the theoretical expectation of 10^15^ [1]. The maturation of the sequences is localized in distinct organs. Differences in the relative abundances, i.e. by comparing subtypes of cells, activation states, tissues or time points, are hence accredited to systemic signaling. The study of these promises novel insights in basic research on the regulation of immune processes and with direct links to autoimmune disorders and oncology, with good prospects for early diagnoses and therapy monitoring [2].

Next generation sequencing (NGS) technologies allow to quantify B and T cell receptor diversity. Under the influence of steadily improving sequencing technologies and decreasing costs, the amount of available raw data rapidly increases. As a consequence, software is required that provides fast and comprehensive means for their analysis to answer both general and highly specific questions.

Several algorithms and tools are available to process raw NGS data of multiple samples. For example, MiXCR is a well-accepted tool to extract BCR and TCR clonotypes from raw NGS data [3]. The recently developed tool LymAnalyzer provides a GUI but does not include options for all sub-tasks in data processing, e.g. paired-end merging [4].

For more conclusive comparisons of BCR/TCR data further analyses are required. The scientific community provides partial implementations of the complete workflow, e.g. tcR, gplots, RCircos and descriptive statistics [5, 6], but these software libraries are complex and demand advanced programming skills. This creates barriers for new users who are not familiar with command line interfaces and programming.

**Fig. 1:**
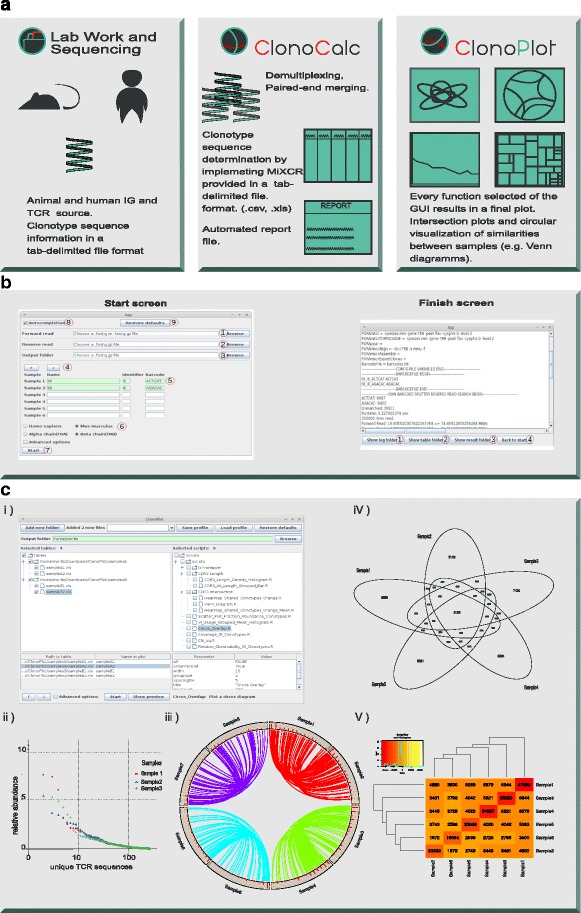
ClonoCalc and ClonoPlot provide a GUI to guide the scientist through a complete workflow for generating publication-ready figures from NGS raw data: **a** Animal and human TCR and IG sequences are input, ClonoCalc pre-processes FASTQ files. ClonoPlot creates final plots using R scripts for data comparison and visualization. **b** Screenshots from ClonoCalc application. From the start screen, a button opens a file dialog, where FASTQ-files can be chosen. Parameters for sub-tasks of data preprocessing can be chosen from buttons depicted on the right. A log file to review the analysis will be generated automatically and made available on the finish screen. **c** ClonoPlot’s user interface *(i)* is divided into two sides: on the left, folders can be chosen from the working directory and will be added to the treeview. On the right, various functions can be selected, each of which produces a plot. Examples of resulting plots include frequency plots *(ii)*, circular visualization of similarities between samples *(iii)*, and CDR3 intersection plots including Venn diagrams and heatmaps *(iV and V)*

We therefore developed ClonoCalc and ClonoPlot, two open source programs that wrap a selection of widely-accepted freely available tools in order to simplify and streamline the analysis workflow for BCR/TCR repertoire studies. These new tools make these analyses accessible to users with less advanced programming skills. A newly developed graphical interface guides the user through the process from parsing raw data to creating diagrams for comparative and statistical analyses. The whole workflow is summarized in Fig. 1 a. Advanced users can adapt and extend the applications due to the adoption of a modular architecture. An additional batch mode permits high throughput execution of a defined analysis protocol without requiring the user to launch the GUI.

## Implementation

### ClonoCalc

ClonoCalc provides a GUI for the sub-tasks of processing raw NGS data by wrapping existing 3rd-party implementations of multiple algorithms (Fig. 1 b, left). The wrapped tools are started from within BASH shell scripts which may be amended by advanced users without a need for a development environment. ClonoCalc’s Java front-end handles only the user input, while the main processing is passed to the shell scripts to execute the individual analysis steps and to aggregate their output. ClonoCalc ships with interfaces to a series of current well-established tools and comprises the following features:

- Demultiplexing of samples: ClonoCalc first splits the raw data (expected in FASTQ format) and classifies the individual reads according to their barcode by wrapping the FASTX Barcode Splitter [7] (Fig. 1 b, start screen (5)). This process needs to suit the wet-lab protocol and limitations of the Illumina demultiplexer led to the employment of the FASTX Barcode Splitter [7] for preprocessing. The user selects input and output files for the analysis and enters the barcode to identify specific samples. A readable name can be chosen for every sample and the user is supported by an auto completion and error correction system. Additional parameters for accurate identification of the barcode can be selected from the GUI. This allows for a perfect matching of the barcode while expecting a downstream shift of its location in the sequence (Fig. 1 b).
- Clonotype determination: Raw NGS data is processed by wrapping the tool MiXCR to perform paired-end read merging (if using the Illumina system). MiXCR extracts human or animal BCR and TCR clonotypes providing corrections of erroneous sequences introduced by NGS [3] (Fig. 1 b).
- Automated documentation: A report of the data analysis process is automatically created with every workflow invocation (Fig. 1 b, right) to facilitate the review and report of the observations made. The call parameters (options) can be modified and will be stored for later reproduction of the analysis. A log file extends the report with detailed information on the performed run, including the configuration parameters and the output of MiXCR (Fig. 1 b).
- Flexibility: Other back-ends may be integrated by the user, substituting MiXCR or the demultiplexer by adjusting the respective call script.

### ClonoPlot

ClonoPlot provides a user-friendly GUI to visualize observations in comparative BCR and TCR repertoire analysis (Fig. 1 c). It removes any requirement for users to become familiar with the programming language R in order to create high-quality figures (Fig. 1 c, ii–V.). The desired figure types can be selected from within the GUI. ClonoPlot offers the following features:

- A tab-delimited file format with clonotype sequence information, e.g. their DNA sequence, its translation to amino acids and their abundances, is imported from ClonoCalc. The table may be offered in CSV or XLS format, similar to the output format of the MiXCR software.
- Primary descriptive analyses are performed by wrapping the tcR package [5].
- Comparative statistical analyses are offered with support of the RCircos package [6], Venn diagrams generated by the gplots package and custom routines to assess the differences between samples.
- ClonoPlot stores the program state and supports profiles to allow for quick transitions between multiple projects or configurations. A preview mode allows the user to review and save specific visualizations. A batch mode allows multiple plots to be created and saved at once.
- The set of available diagram types can be extended, if required, to meet local demands. To do so, the user provides a custom R script file in ClonoPlot’s working directory. ClonoPlot will automatically read this script and makes the new plot type available in the GUI.

ClonoPlot’s Java front-end communicates with the R extension Rserve [8] which itself maintains the underlying R session (see Additional file 1: Figure S1). Furthermore, the R back-end brings the full language support including all packages and the ability to start third-party executables for integration into the visualization process. The Java application parses R documentation headers from the script files and displays information on available functions and their arguments in the GUI. Moreover, errors occurring in the R back-end will be displayed in the GUI, allowing scripts to react to possible user errors and return comprehensible error messages. To ensure reproducibility the batch mode provides the option to save the complete R workspace and all executed commands. Consequently, every step leading to the final plot has full provenance and can be audited and verified.

## Results and discussion

NGS technologies enable deep analysis of repertoires and their diversity and facilitate the investigation of immune functions in health and disease. The available data is huge and analysis is not practical without dedicated tools. Therefore there is a strong demand for bioinformatic pipelines for comparative analyses of BCR and TCR repertoires.

The approach we developed has been accepted by local practical colleagues both as a way to reduce their workload and to become familiar with the underlying technology. It combines well tested and robust tools for primary (from FASTQ format to clonotype determination) and advanced BCR and TCR analysis (comparative and statistical approaches) and extends several functions.

We provide two executables to support the common workflow in a lab environment. First, ClonoCalc works on data as directly retrieved from the sequencer to identify the clonotypes. This first step considerably reduces the data size for downstream comparative analyses with ClonoPlot. ClonoCalc is expected to be executed only once per project by a more technical person on powerful hardware. ClonoPlot is more likely to be executed with a scientific question in mind and analyses rerun with parameters changed. Together, ClonoCalc and ClonoPlot provide a complete analysis pipeline (see comparison in Table 1).

**Table 1:** Feature comparison of ClonoCalc/ClonoPlot, MiXCR [3], LymAnalyzer [4], tcR [5] and VDJtools [9]

	ClonoCalc/ClonoPlot	MiXCR	LymAnalyzer	tcR & VDJtools
GUI		✕	✔	✕
Demultiplexing	✔ (wraps FASTX)	✔	✕	✕
Paired-end merging	✔	✔	✕	✕
TCR/Immunoglobulins	✔ (wraps MiXCR)	✔	✔	✕
CDR3 Extraction, including error correction	✔ (wraps MiXCR)	✔	✔	✕
V, D and J gene alignment	✔ (wraps MiXCR)	✔	✔	✕
Evaluation of relative/absolute frequency of clonotypes	✔	✕	✕	✔
Statistics (CDR3 length, VJ gene usage)	✔ (wraps tcR)	✕	✕	✔
Descriptive statistics providing means and standard deviation (CDR3 length, VJ gene usage)		✕	✕	✕
CDR3 intersection plots, like heatmaps and	✔	✕	✕	✔
Venn diagrams		✕	✕	✕
Circular visualization of similarities and differences		✕	✕	✕
Automated documentation of all analysis steps		✕	✕	✕

Novel contributions from ClonoPlot/ClonoCalc are highlighted in green

Table 1 provides a feature comparison of ClonoCalc and ClonoPlot with a selection of similar tools from a broader overview in the supplement to the paper accompanying VDJtools [9]. ClonoCalc provides a GUI that combines all sub-tasks of raw data processing. ClonoPlot in particular wraps existing tools such as the tcR package for visualization and contributes additional functions for advanced and comparative analyses of BCR/TCR repertoires as summarized in Table 1.

We found other GUI-enabled tools such as LymAnalyzer lacking in features for analysis or pre-processing. Command-line tools such as MiXCR and VDJtools provide primary and descriptive analyses for BCR and TCR repertoires but lack a GUI [3, 4, 9].

ClonoCalc and ClonoPlot together with accompanying documentation can be freely downloaded. Both tools can be executed on Linux, MacOS and Windows. The applications are modular and as such advanced users can adapt it to the latest developments in the field or incorporate additional features based on their existing in-house expertise. An alternative to the direct implementation using Java and incorporating R would have been the deployment of custom workflows built upon generic workflow engines. Due to the relative simplicity of the underlying processes, we considered this approach unduly complicated.

Web-based applications come with the advantage that no local software has to be installed and that multiple projects can be run in parallel. However, data privacy considerations together with the multi-user support required of a central server solution led us to prefer the development of a tool suitable for installation on a local computer.

## Conclusion

Our two new introduced programs implement a complete workflow for the analysis of B and T cell repertoires from raw data in a way that is more readily accessible to scientist and technicians. These tools enable immunologists unfamiliar with computational languages to investigate immune repertoires and produce publication-ready figures. ClonoCalc expects raw data for primary analyses to determine human or animal BCR and TCR clonotypes. Subsequently, ClonoPlot provides descriptive and comparative statistical analysis for visualization. ClonoCalc and ClonoPlot provide a GUI, reducing barriers for less experienced users and providing a guide to scientists.

## Availability and requirements

**Project home page:** https://bitbucket.org/ClonoSuite/clonocalc-plot.The source code contains self generated sample raw sequence data from murine spleen.**Operating systems:** Linux, MacOS X, Windows**Programming language:** R, Java.**License:** GNU GPL v3

## Additional file

Additional file 1
**Figure S1.** Component diagram shows the software architecture of ClonoPlot. (PDF 22 kb)

## Abbreviations

BCR: B cell receptor
CDR: Complementarity determining region
IG: Immune globulin
GUI: Graphical User Interface
NGS: Next generation sequencing
TCR: T cell receptor
