# Supplementary material for: ClonoCalc and ClonoPlot: immune repertoire analysis from raw files to publication figures with graphical user interface
Source: BMC Bioinformatics. 2017 Mar 11;18:164. doi: 10.1186/s12859-017-1575-2 (PMC5346239; doi:10.1186/s12859-017-1575-2)
Supplement: Additional file 1 — Figure S1. Component diagram shows the software architecture of ClonoPlot. (PDF 22 kb) [file 12859_2017_1575_MOESM1_ESM.pdf]

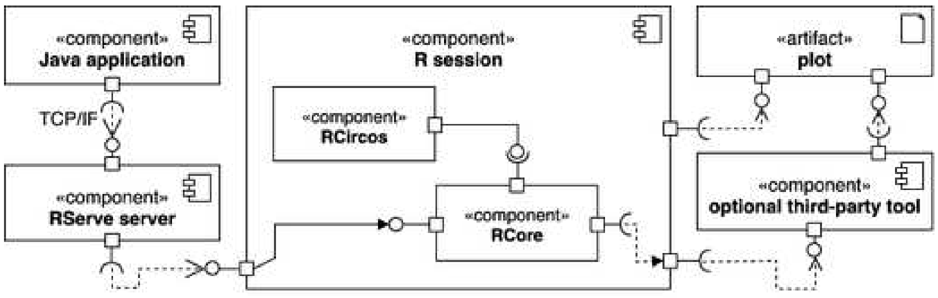

Figure S1 Component diagram shows the software architecture of ClonoPlot. The Java and R code base allows for execution of ClonoPlot on all major operating systems. The user-facing Java GUI application communicates with the server component of the R library RServe. The RServer forwards the R-language expressions to the R session on behalf of the Java application. The R session in turn evaluates the expressions and calls additional libraries such as RCircos. Possible results are plots,data-tables or the outputs of third-party tools.
